# Crizotinib-induced hyperlipidemia in advanced lung adenocarcinoma: A case report and literature review

**DOI:** 10.1097/MD.0000000000047276

**Published:** 2026-01-23

**Authors:** Shuai Ren, Ruifang Chen

**Affiliations:** a Department of Respiratory and Critical Care Medicine, Shanghai Public Health Clinical Center, Shanghai, China.

**Keywords:** crizotinib, hyperlipidemia, lung adenocarcinoma, ROS1 fusion gene-positive

## Abstract

**Rationale:**

Crizotinib is a multi-target small molecule tyrosine kinase inhibitor that specifically targets anaplastic lymphoma kinase, mesenchymal-epithelial transition factor/hepatocyte growth factor receptor, and c-ROS oncogene 1 (ROS1). Its primary application in clinical settings is for the treatment of advanced non-small-cell lung cancer characterized by anaplastic lymphoma kinase-positive and/or ROS1 fusion gene-positive status. Hyperlipidemia is a rarely documented adverse reaction to crizotinib, with its underlying mechanisms and clinical management strategies remaining unclear.

**Patient concerns:**

Significant dyslipidemia was observed in a 70-year-old female with advanced, ROS1 fusion-positive lung adenocarcinoma during crizotinib therapy.

**Diagnoses:**

This case report presents a patient diagnosed with advanced lung adenocarcinoma harboring an ROS1 fusion gene, who subsequently developed severe hyperlipidemia during treatment with the targeted agent crizotinib.

**Interventions:**

A combined strategy of crizotinib dose reduction and concomitant lipid-lowering therapy was initiated to address the hyperlipidemia, aiming to maintain control of the primary malignancy while managing the adverse effect.

**Outcomes:**

Following a reduction in the dosage of crizotinib and the implementation of proactive lipid-lowering interventions, both the malignancy and the associated adverse effects were effectively controlled.

**Lessons:**

The condition exhibited a typical positive rechallenge feature of “discontinuation and recurrence,” providing critical clinical evidence to establish this causal relationship. The case aims to enhance the understanding of crizotinib’s metabolic side effects, underscores the importance of dynamic lipid monitoring, and offers empirical support for the standardized management of such rare adverse reactions.

## 1. Introduction

The proto-oncogene c-ROS oncogene 1 (ROS1) encodes a receptor tyrosine kinase whose physiological function in humans remains largely unclear.^[1]^ Extensive research has illuminated its involvement in various malignancies, including non-small-cell lung cancer (NSCLC), cholangiocarcinoma, gastric cancer, ovarian cancer, and glioblastoma multiforme, where the oncogenic ROS1 receptor tyrosine kinase is activated through chromosomal rearrangement.^[2-6]^ These rearrangements result in the fusion of a segment of ROS1 – retaining the complete tyrosine kinase domain – with one of twelve distinct partner proteins, thereby triggering the continuous activation of the ROS1 fusion kinase and propelling cellular transformation.^[7,8]^ It is important to note that approximately 1% to 2% of patients with NSCLC present with ROS1 gene rearrangements, while a comparable 3% to 7% exhibit anaplastic lymphoma kinase (ALK) rearrangements.^[9,10]^ Crizotinib, an inhibitor targeting ALK, ROS1, and mesenchymal-epithelial transition factor/hepatocyte growth factor receptor, has demonstrated potent antitumor efficacy.^[9]^ In 2024, the Chinese Society of Clinical Oncology recommended crizotinib, alectinib, brigatinib, lorlatinib, ensartinib, erdafitinib, and ceritinib for the treatment of ALK-positive NSCLC patients, marking crizotinib and entrectinib as the preferred first-line targeted therapies for individuals with ROS1 fusion gene-positive disease. Crizotinib is commonly associated with adverse effects such as diarrhea, nausea, vomiting, edema, fatigue, hepatic dysfunction, and neutropenia.^[11]^ Although hyperlipidemia and hypercholesterolemia occur less frequently, lorlatinib is primarily associated with lipid abnormalities.^[11]^ This report details the case of a patient who experienced severe hyperlipidemia as a consequence of crizotinib treatment.The participant provided informed written consent after being fully briefed on the study procedures.

## 2. Case presentation

The subject of this case study is a 70-year-old female patient, with a height of 155 cm and a weight of 60 kg. She has a documented history of hypertension spanning 10 years, which has been effectively managed. The patient has no history of tobacco use and lacks a familial predisposition to neoplasms. On July 19, 2023, she presented to our institution with complaints of severe chest tightness and exertional dyspnea that had persisted for over a week. A physical examination yielded no significant findings.

**Figure 1.**
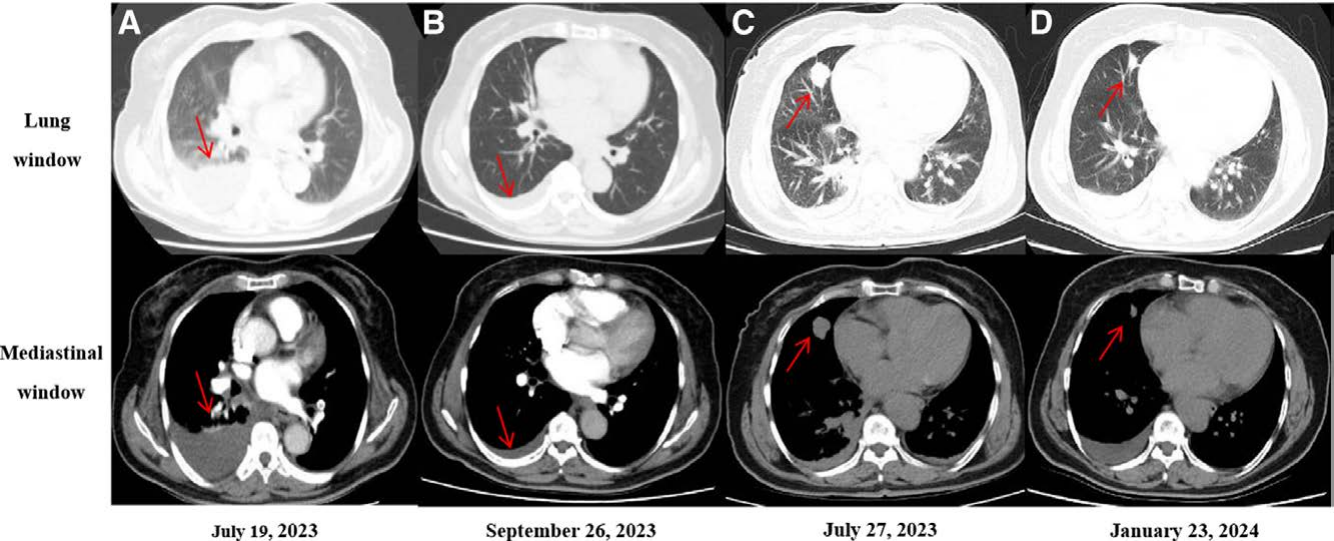
A pretreatment, July 19, 2023: (A) CTA of the pulmonary artery demonstrated the presence of emboli in the left pulmonary artery, alongside partial consolidation and atelectasis in the middle and lower lobes of the right lung, as well as a right-sided pleural effusion. (B) Post-anticoagulant therapy, September 26, 2023: subsequent to the initiation of anticoagulant therapy, an evaluation of the pulmonary artery CTA revealed residual emboli in the left pulmonary artery, persistent partial consolidation in the lower lobe of the right lung, with no significant changes noted, and ongoing pleural effusion on the right side. (C) Pre-targeted therapy, July 27, 2023: prior to the commencement of targeted therapy, a chest CT scan revealed a space-occupying lesion in the right lung, measuring approximately 23 mm by 18 mm. (D) Six months post-targeted therapy, January 23, 2024: after 6 months of targeted therapy, a follow-up chest CT scan showed a reduction in the size of the pulmonary lesion to approximately 15 mm by 8 mm. CT = computed tomography, CTA = computed tomography angiography.

Subsequent pulmonary artery computed tomography angiography revealed the presence of thrombi in the left upper and lower lobe pulmonary arteries, with suspected scattered thrombi in the right upper and lower lobe pulmonary arteries. Additionally, there were indications of partial consolidation and atelectasis in the right middle and lower lobes, right pleural effusion, and enlargement of mediastinal lymph nodes (Fig. 1A). The patient was commenced on low molecular weight heparin (4100 AXaIU every 12 hours, administered subcutaneously) for anticoagulation. A thoracentesis was performed under B-ultrasound guidance for fluid aspiration, and the aspirated fluid was subsequently sent for laboratory analysis and pathological evaluation.

On July 21, 2023, the analysis of the pleural effusion revealed elevated levels of carcinoembryonic antigen at 65.2 ng/mL, cancer antigen 125 at 843 U/mL, cancer antigen 15-3 exceeding 300 U/mL, and lactate dehydrogenase at 729 U/L. A chest computed tomography scan conducted on July 27, 2023, indicated a high probability of a malignant tumor located in the medial segment of the right middle lobe, measuring approximately 23 mm × 18 mm, accompanied by mediastinal lymph node enlargement; small pleural effusions were noted bilaterally, with right-sided drainage (Fig. 1C). The pathological examination of the thoracentesis fluid confirmed a diagnosis of lung adenocarcinoma. Immunohistochemical analysis demonstrated positive staining for CK7, TTF-1, and napsin A, while being negative for CK5/6, P40, SYN, among others, with mesothelial cells showing positive staining for calretinin. Genetic testing identified a SLC34A2-ROS1 fusion mutation. The patient was diagnosed with right lung adenocarcinoma (cT1cN2M1a, stage IVA, ROS1 fusion gene-positive). In accordance with established treatment guidelines, crizotinib (250 mg administered twice daily) was prescribed as the first-line targeted therapy.

A follow-up pulmonary artery computed tomography angiography performed on September 26, 2023, indicated a reduction in thrombus formation within the anterior basal segment of the left lower lobe pulmonary artery, with some thrombi resolving compared to the imaging conducted on July 19, 2023; however, partial consolidation in the right middle and lower lobes and right pleural effusion persisted (Fig. 1B). On January 23, 2024, a follow-up chest computed tomography demonstrated a gradual decrease in the size of the lesion in the medial segment of the right middle lobe to 15 mm × 8 mm, with mediastinal lymph nodes appearing smaller than previously observed. The therapeutic response was classified as a partial response (Fig. 1D), and crizotinib targeted therapy was continued. During a follow-up visit on March 15, 2024, the patient’s lipid profile revealed a triglyceride level of 11.41 mmol/L and a cholesterol level of 7.25 mmol/L.

**Figure 2.**
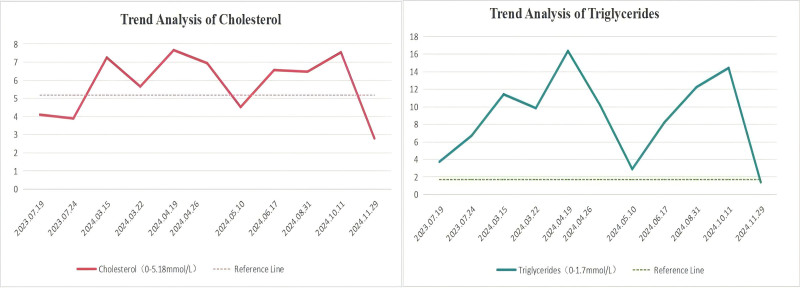
Evolution of blood lipid profiles in patients.

According to the “Multidisciplinary Expert Consensus on the Clinical Management of Hypertriglyceridemia,” the patient’s condition was classified as an extremely severe elevation of triglycerides. In accordance with established guidelines for the treatment of hyperlipidemia, the patient was advised to follow a low-fat diet and was prescribed rosuvastatin at a dosage of 10 mg nightly for lipid regulation. By March 22, 2024, the patient’s triglyceride levels had decreased to 9.84 mmol/L, and cholesterol levels had reduced to 5.65 mmol/L, prompting a recommendation to continue the medication. However, the patient discontinued the medication for a period of 2 weeks, resulting in a significant increase in triglycerides to 16.36 mmol/L and cholesterol to 7.66 mmol/L. To further manage lipid levels, fenofibrate at a dosage of 100 mg daily was introduced. Given the patient’s medical history, it was hypothesized that crizotinib may have contributed to the development of mixed hyperlipidemia; consequently, the crizotinib dosage was adjusted to 250 mg daily to facilitate ongoing oral targeted therapy. The patient intermittently utilized lipid-lowering medications, with lipid levels demonstrating an increase upon self-discontinuation (Table 1, Fig. 2). Since October 2024, the patient has consistently adhered to the prescribed regimen of rosuvastatin and fenofibrate. By November 29, 2024, the patient’s lipid levels had returned to normal. A comprehensive evaluation at this time indicates that the patient’s lung lesions remain stable.

**Table 1:** Sequential variations in patient blood lipid levels.

Inspection date	Total cholesterol (0–5.18 mmol/L)	Triglycerides (0–1.7 mmol/L)	High-density lipoprotein cholesterol (1.29–1.55 mmol/L)	Alanine aminotransferase (7–40 U/L)	Aspartate aminotransferase (13–35 U/L)
19.07.2023	4.10	***3.72***	0.93	14.00	13.00
24.07.2023	3.89	***6.69***	0.87	33.90	***40.00***
15.03.2024	***7.25***	***11.41***	0.75	15.00	15.00
22.03.2024	***5.65***	***9.84***	0.61	26.00	21.00
19.04.2024	***7.66***	***16.36***	0.73	24.00	22.00
26.04.2024	***6.94***	***10.17***	0.79	21.00	18.00
10.05.2024	4.52	***2.89***	1.04	14.00	19.00
17.06.2024	***6.56***	***8.20***	0.77	20.10	19.00
31.08.2024	***6.47***	***12.23***	0.54	17.30	18.80
11.10.2024	***7.54***	***14.42***	0.58	21.00	17.00
29.11.2024	2.79	1.39	0.85	35.50	33.10

Data highlighted in both bold and italics represent elevated values.

## 3. Discussion

In this case study, the patient was diagnosed with pulmonary adenocarcinoma, and genetic analysis identified an SLC34A2-ROS1 fusion mutation. The conclusive diagnosis was stage IVA right lung adenocarcinoma (cT1cN2M1a) characterized by the presence of a positive ROS1 fusion gene. Following established clinical guidelines, the patient commenced treatment with crizotinib, an oral targeted therapy. Throughout the patient’s treatment trajectory, a thorough assessment indicated a partial therapeutic response (Fig. 1C, D).

Prior to the initiation of crizotinib, the patient displayed a mildly elevated lipid profile (Table 1). The patient in this case had mildly elevated blood lipid levels prior to initiation of crizotinib (Table 1). Starting from August 3, 2023, the patient began taking crizotinib, and during follow-up, significant increases in total cholesterol and triglycerides were gradually observed (Table 1). In response, rosuvastatin and fenofibrate were administered for lipid control, and the dose of crizotinib was appropriately reduced. During the course of treatment, the patient discontinued the lipid-lowering agents on several occasions upon observing a decrease in lipid levels, which subsequently led to repeated sharp rises in blood lipids. This pattern effectively constituted a de facto “drug rechallenge”: withdrawal of the lipid-lowering therapy (cessation of exposure) resulted in the reemergence of hyperlipidemia, while resumption of the medication brought lipid levels back under control. This dynamic process strongly supports a clear temporal association and causal relationship between the observed severe hyperlipidemia and the administration of crizotinib, consistent with the evaluation criteria for adverse drug reactions.

Crizotinib is known to be associated with gastrointestinal side effects, including diarrhea, nausea, and vomiting, as well as other adverse effects such as edema, fatigue, hepatic dysfunction, and neutropenia. Among the various targeted therapies for ALK-positive cancer, lorlatinib is most frequently associated with dyslipidemia.^[11]^ The most frequently reported adverse reaction to encorafenib is taste disturbance, with fatigue, dizziness, nausea, and diarrhea also commonly observed.^[12]^ In a separate study, weight gain and neutropenia were the most common adverse reactions, while neurological disorders and cardiac conditions represented the primary serious treatment-related adverse events.^[13]^ For alectinib, commonly reported low-grade (1–2) adverse events include constipation, nausea, vomiting, diarrhea, myalgia, and peripheral edema.^[14]^ The most frequent grade 3 to 4 adverse events were elevated liver enzymes (approximately 3% of patients), increased blood bilirubin (3.2%), and anemia (2%).^[14]^ Brigatinib is often associated with cough, hypertension, rash, and elevations in creatine kinase, lipase, and amylase.^[15]^ It is noteworthy that lorlatinib was the only agent among them associated with a significant effect on blood lipid levels. In a phase 3 trial comparing lorlatinib with crizotinib in advanced ALK-positive NSCLC, lorlatinib demonstrated a significantly higher incidence of dyslipidemia.^[16]^ Hypercholesterolemia occurred in 70% of patients on lorlatinib versus 4% on crizotinib; hypertriglyceridemia affected 64% versus 6%, respectively.^[16]^ Notably, among the 142 crizotinib-treated patients, only 5 developed hypercholesterolemia (all grade 1) and 8 experienced hypertriglyceridemia (grades 1–2).^[16]^ Importantly, treatment interruption rates due to adverse events were comparable between the 2 groups (7% lorlatinib; 9% crizotinib).^[16]^ Management of lorlatinib-induced dyslipidemia does not typically require treatment discontinuation unless severe hypercholesterolemia (>12.93 mmol/L) or hypertriglyceridemia (>11.3 mmol/L) occurs, as it is generally controlled with dose modification and/or lipid-lowering therapy.^[17]^ While severe crizotinib-induced hyperlipidemia has not been previously reported and no specific guidelines exist, we observed a case in which triglyceride levels rose sharply to 16.36 mmol/L during targeted therapy. Nevertheless, through consistent lipid-lowering medication and dose reduction, the patient’s lipid levels were successfully stabilized within the normal range.

Literature review suggests that crizotinib, metabolized by cytochrome P450 3A (CYP3A), can interact with CYP3A4 inhibitors. This co-administration may raise its plasma levels, potentially intensifying pharmacological effects or adverse events.^[18]^ Early clinical studies established that crizotinib undergoes extensive phase I metabolism primarily via CYP3A.^[19]^ Concomitant use with ketoconazole (a CYP3A inhibitor) or rifampicin (a CYP3A inducer) has been shown to significantly alter crizotinib exposure by affecting its pharmacokinetics.^[18,20]^ Similarly, lorlatinib acts as both a substrate and an inducer of CYP3A. Prior evidence indicates that co-administration should be avoided with moderate CYP3A inducers, strong CYP3A inhibitors, and narrow therapeutic index CYP3A substrates.^[17]^ Specifically, CYP3A inhibitors can markedly increase lorlatinib plasma concentrations, whereas CYP3A inducers may reduce its efficacy and induce severe hepatotoxicity.^[17]^

These studies have delineated the metabolic profiles and drug interaction potentials of crizotinib and lorlatinib, highlighting both shared and distinct characteristics. Building on this foundation, future research should further analyze these aspects to elucidate the underlying mechanisms of therapy-associated lipid abnormalities.

Beyond this, the patient demonstrated simultaneous elevations in blood cholesterol and triglyceride levels, a condition known as mixed hyperlipidemia. This condition can be classified into primary and secondary forms. Primary hyperlipidemia is primarily linked to genetic mutations, frequently exhibiting familial clustering and a significant genetic predisposition. However, the patient indicated an absence of any familial history of hyperlipidemia. In contrast, secondary hyperlipidemia may result from systemic disorders such as diabetes, obesity, hypothyroidism, or nephrotic syndrome, or it may be triggered by specific medications. Regardless of the underlying cause, any disruption in the critical enzymes involved in lipid synthesis, lipoprotein metabolism, or receptor-mediated degradation can lead to dyslipidemia. The liver is integral to lipid metabolism, and its dysfunction can result in increased levels of both triglycerides and cholesterol.^[21]^

In 2021, an expert panel from the Integrated Oncology Cardiology Branch of the Chinese Anti-Cancer Association conducted a synthesis of clinical research regarding the impact of antitumor agents on lipid profiles. Their findings indicated that these pharmacological agents can induce dyslipidemia through the modulation of enzymes involved in lipid metabolism, such as lipoprotein lipase and hepatic triglyceride lipase. This modulation occurs via alterations in the expression of apolipoproteins, variations in adiponectin levels, or the induction of insulin resistance, which can impair pancreatic β-cell function and reduce gonadal function.^[22]^

Throughout the patient’s treatment trajectory, critical physiological parameters – including blood glucose levels, thyroid function, gonadal function, urinalysis, and albumin levels – have exhibited notable stability. These metrics serve as robust indicators of the patient’s overall health status. It is widely recognized that crizotinib is predominantly metabolized by the liver. However, the potential consequences of its hepatic effects on the apolipoprotein composition ratio – such as increased low-density lipoprotein synthesis, decreased high-density lipoprotein synthesis, reduced lipoprotein lipase activity, and diminished hepatic triglyceride lipase activity – thereby leading to disruptions in lipid synthesis and metabolism, necessitate further comprehensive investigation.

Meanwhile, this study is subject to several limitations. Its nature as a single-center case report limits the statistical power and generalizability of the findings. Furthermore, the follow-up period of over 1 year, while substantial, may be inadequate to assess the long-term effects of the drug on lipid levels. Future research requires prolonged monitoring to track the ongoing changes in lipid parameters.

## 4. Conclusions

In the medical literature, we present a novel case of a patient who developed significant hyperlipidemia following treatment with crizotinib. Through the consistent administration of lipid-lowering agents and meticulous adjustments to the crizotinib dosage, we achieved not only a substantial reduction in lipid levels but also a notable inhibition of the progression of pulmonary lesions, thereby illustrating a dual therapeutic success. This case provides important clinical insights for the management and treatment of hyperlipidemia in patients receiving crizotinib. Lorlatinib is frequently associated with hyperlipidemia, while crizotinib only rarely induces severe dyslipidemia. In this specific case, however, a clear correlation has been established between the patient’s hyperlipidemia and crizotinib administration. Consequently, we recommend enhanced lipid monitoring during crizotinib therapy, advocating for patients to undergo at least 1 lipid assessment per month. Furthermore, we encourage further investigation into the underlying mechanisms of drug-induced dyslipidemia, as this will contribute to a more comprehensive understanding of these phenomena and inform future treatment approaches.

## Author contributions

**Data curation:** Ruifang Chen.

**Funding acquisition:** Ruifang Chen.

**Supervision:** Shuai Ren.

**Writing – original draft:** Ruifang Chen.
